# Associations Between Successful Home Discharge and Posthospitalization Care Planning: Cross-Sectional Ecological Study

**DOI:** 10.2196/56091

**Published:** 2024-12-12

**Authors:** Naoki Takashi, Misaki Fujisawa, Shosuke Ohtera

**Affiliations:** 1 Department of Health Economics Center for Gerontology and Social Science Research Institute, National Center for Geriatrics and Gerontology Aichi Japan

**Keywords:** health services research, health policy, quality of care, access to care, outcome assessment, public health, health service, accessible, accessibility, care coordination, health outcome, surveillance, regional disparities, nonstandardized care, nonstandardization, hospital discharge, hospital care, analysis, Japan, older adults

## Abstract

**Background:**

Effective discharge planning is crucial for successful care transitions, reducing hospital length of stay and readmission rates. Japan offers a financial incentive to enhance the coordination of posthospitalization care planning for patients with complex needs. However, the national impact of this incentive remains unclear.

**Objective:**

This study aimed to (1) assess the association between the number of claims submitted for discharge planning, as an indicator of the provision of posthospitalization care planning, and key health care outcomes, including discharges to home, 30-day readmissions, length of stay, and medical expenditures at the prefectural level in Japan, and (2) to describe regional differences in the provision of posthospitalization care planning and explore associated factors.

**Methods:**

This ecological study used prefectural-level data from fiscal year 2020. Claims submitted for discharge planning were used as indicators that posthospitalization care planning was provided. Supply-adjusted standardized claim ratios (SCRs) were calculated using data from the Seventh National Database of Health Insurance Claims, to evaluate and compare the number of claims across 47 prefectures in Japan, accounting for differences in population structure. Key outcomes included discharges to home, 30-day readmissions, length of stay, and medical expenditures. Multivariate negative binomial regression models assessed associations between SCRs and outcomes, adjusting for socioeconomic covariates. In addition, regional differences in the provision of posthospitalization care planning and associated factors were analyzed using the Mann-Whitney *U* test. Prefectures were divided into 3 groups (low, medium, and high) based on tertiles of each factor, and supply-adjusted SCRs were compared across these groups.

**Results:**

The ratio of the minimum to maximum supply-adjusted SCR was 10.63, highlighting significant regional variation. Higher supply-adjusted SCRs, indicating more frequent provision of posthospitalization care planning, were associated with an increase of 9.68 (95% CI 0.98-18.47) discharges to home per 1000 patients for each SD increase in supply-adjusted SCR. Several factors contributed to regional differences in the supply-adjusted SCR for posthospitalization care planning. A higher supply-adjusted SCR was significantly associated with a greater number of nurses per 100 hospital beds (median SCR in low, medium, and high groups: 0.055, 0.101, and 0.103, respectively); greater number of care manager offices per 100 km^2^ of habitable area (0.088, 0.082, and 0.116); higher proportion of hospitals providing electronic medical information to patients (0.083, 0.095, and 0.11); lower proportion of older adults living alone (0.116; 0.092; 0.071); and higher average per capita income (0.078, 0.102, and 0.102).

**Conclusions:**

The provision of posthospitalization care planning is associated with an increased likelihood of discharge to home, underscoring its importance in care transitions. However, significant regional disparities in care coordination exist. Addressing these disparities is crucial for equitable health care outcomes. Further research is needed to clarify causal mechanisms.

## Introduction

Hospital discharge represents a critical transition in patient care. Shifting care settings and providers frequently leads to disruptions in care continuity, contributing to adverse events and negative health outcomes [1,2]. This is particularly concerning for older adults, who often have multiple chronic conditions and functional impairments requiring ongoing and coordinated care. Buurman et al [3] underscore this vulnerability, showing that older patients face a heightened risk of functional decline and mortality following hospital discharge. In addition, Forster et al [2] have identified that 19% of patients experience adverse events after discharge, with an estimated 30% of these events being potentially preventable. These findings highlight the critical need for improved transitional care strategies to mitigate risks and enhance patient safety.

Discharge planning is a critical component of transitional care [4,5] and includes multiple components, for example, the initiation of discharge planning upon hospital admission; the employment of a multidisciplinary team for comprehensive assessment; involvement with patients and their caregivers; shared decision-making; and collaboration with postdischarge health care, long-term care, and social service providers [6]. Recent meta-analyses indicate that effective discharge planning significantly reduces hospital length of stay mean difference: –0.73 days and –0.71 days) and readmission rates (relative risk: 0.89 and 0.78) [6,7].

Integrating effective strategy into the broader health care delivery system is essential. Financial incentives may serve as effective mechanisms to standardize interventions and improve care quality [8,9]. In Japan, health insurance provides financial incentives for hospital discharge planning when staff, including nurses and social workers, coordinate medical and nursing services after discharge [10,11]. This incentive requires hospitals to coordinate posthospitalization care planning for patients with complex care needs, as well as to establish and maintain collaboration with long-term care facilities and social care institutions that the patients use after discharge to ensure seamless transitions and continuity of care [10].

In 2016, Japan revised the incentive for hospital discharge planning to enhance the coordination of posthospitalization care. This change was part of a broader effort to develop a system that provides comprehensive community-based care and services, enabling patients to continue living in their familiar communities for as long as possible [10]. While discharge destinations in the community for older patients include homes, long-term care facilities, and nursing homes, many prefer to return home [12]. Discharge to home remains a key aspect of patient-centered care, reflecting the importance of aligning health care services with patient preferences and promoting quality of life.

In other high-income countries, the impact of policy measures, such as setting incentives, on patient outcomes has been evaluated at the national level. For example, in Sweden, it was reported that the Care Coordination Act, which mandated the creation of a standardized discharge planning process, may have reduced hospital stays [13]. However, in Japan, such evaluations remain limited. Although some municipal-level analyses using administrative databases have examined the effectiveness of this incentive for hospital discharge planning [11,14], its impact at the national level remains unclear.

We conducted this study to gain insights into health care policy development by understanding national trends in the impact and use of the incentive for hospital discharge planning, aimed at enhancing posthospitalization care planning. This study serves as a milestone for future detailed analyses using individual-level data. It has two primary aims: (1) to examine the association between the provision of posthospitalization care planning and key health care outcomes, including discharges to home, 30-day hospital readmissions, length of hospital stay, and medical expenditures at the prefectural level in Japan, and (2) to describe regional differences in the provision of posthospitalization care planning and explore the factors associated with these variations.

## Methods

### The Incentive for Hospital Discharge Planning in Japan

Health care services in Japan covered by universal health coverage are provided on a fee-for-service basis according to a national fee schedule. All health care facilities, including hospitals and clinics, must adhere to the standardized service processes and reimbursement requirements set by the Ministry of Health, Labour, and Welfare (MHLW). The incentive for hospital discharge planning is included in the national fee schedule, specifically under Nyutaiin Shien Kasan No. 1 (NSK1), which focuses on coordinating posthospitalization care planning and establishing collaboration with long-term care facilities and social care institutions. The standardized process and reimbursement requirements for NSK1 mandate the creation of a discharge plan; the organization of meetings; and the inclusion of the patient, their family, and multidisciplinary providers early in the hospitalization phase. It also requires coordination of posthospitalization care planning and communication with long-term care and social care providers to ensure seamless transitions. Given these requirements, we define a claim for NSK1 as an indication that the hospital is providing posthospitalization care planning.

### Study Design

This ecological study was conducted at the prefectural level. First, we analyzed the associations between the provision of posthospitalization care planning and key health care outcomes in each prefecture, including discharges to home, 30-day hospital readmissions, length of hospital stays, and total medical expenditures. Next, we explored factors that may explain regional differences in the provision of posthospitalization care planning. This manuscript adheres to the Strengthening the Reporting of Observational Studies in Epidemiology (STROBE) statement for reporting cross-sectional studies, ensuring methodological rigor and transparency.

### Ethical Considerations

The study protocol was approved by the Ethics Committee of the National Center for Geriatrics and Gerontology (grant 1752), with a waiver of informed consent due to the use of anonymized, aggregated data that did not include personal information. There was no direct patient involvement in this study.

### Measurements and Data Sources

#### Exposure: Posthospitalization Care Planning

The provision of posthospitalization care planning was measured based on the number of claims for NSK1 in each prefecture in 2020. We assumed that making a claim for NSK1 indicates that posthospitalization care planning was conducted and that collaboration with long-term care facilities and social care institutions was established and maintained. The number of claims in each prefecture was extracted from the Seventh National Database of Health Insurance Claims and Specific Health Checkups of Japan (NDB) open data. The NDB open data are aggregated data derived from the NDB for each fiscal year, starting in 2014. The NDB, developed by the MHLW, is a comprehensive administrative database that includes health insurance claim information covering nearly the entire population of Japan due to the universal health insurance system. The Seventh NDB open data includes claims from April 2020 to March 2021.

The number of claims cannot be directly compared between prefectures because of differences in population structures. Therefore, we calculated the standardized claim ratio (SCR), which enabled a comparison of the number of claims between prefectures with different population structures by adjusting for sex and age [15-17]. This is similar to the standardized mortality ratio. SCR is calculated as follows:



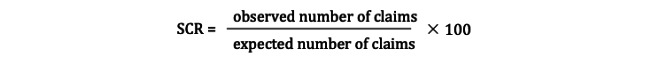




Observed number of claims = the total number of claims in a target prefecture in a year

Expected number of claims = the population of a sex and age group in a target prefecture × the sex- and age-specific claim rate of the sex and age group in all of Japan

The sex- and age-specific claim rate of the sex and age group in all of Japan = ∑ (the specific claims of a sex and age group in all of Japan × the population by sex and age group in all of Japan)

The SCR for each prefecture is set at 100, indicating that the claims adjusted for sex and age in a prefecture are equivalent to those for all of Japan. In other words, an SCR of 100 suggests that the frequency of posthospitalization care planning claims in a prefecture is at the national average level. An SCR higher than 100 indicates a more frequent provision of posthospitalization care planning in the prefecture compared to the national average, whereas an SCR lower than 100 indicates less frequent provision. However, as noted by Taira et al [15], SCRs do not account for health care supply factors within a prefecture. Therefore, a higher SCR could reflect a greater number of hospital beds rather than increased care planning activity. To address this, we calculated a supply-adjusted SCR as follows:



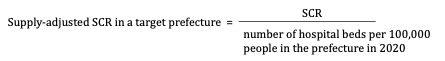



The number of hospital beds per 100,000 people in the prefecture in 2020 was extracted from the Survey of Medical Institutions (Government Statistics Code: 00450021).

#### Outcomes

The outcomes of interest in this study are (1) the number of patients who were discharged from the hospital to home, (2) the number of patients readmitted within 30 days after discharge from a hospital or clinic, (3) the average length of a hospital stay, and (4) medical expenditure in each prefecture in 2020. The former 3 were derived from an open government database, the Patient Survey (Government Statistics Code: 00450022). The latter was extracted from the Estimates of National Medical Care Expenditures (Government Statistics Code: 00450032).

#### Covariates

Previous studies have demonstrated that socioeconomic status is an important factor affecting health care outcomes, such as the destination of hospital discharge, length of hospital stays, and 30-day readmissions [17-20]. Therefore, socioeconomic factors at the prefecture level were considered covariates in this study. Socioeconomic factors included the proportion of older adults living alone and the average income per capita in each prefecture in 2020. The former was calculated using the number of older people living alone and the total number of households in the prefecture in 2020 based on retrieved from the National Census (Government Statistics Code: 00200521). The latter was extracted from the Economic and Social Research Institute Statistics Annual Report on Prefectural Accounts for Average Income [21].

#### Potential Factors Explaining Regional Differences in the Provision of Posthospitalization Care Planning

Factors were selected based on a report by the MHLW [22], previous studies [23,24], and our clinical experience in hospitals. The number of nurses and social workers per 100 hospital beds was used to represent the resources of each hospital, and this statistic was calculated using the number of nurses and social workers in the hospital and the number of beds in the prefecture in 2020. Those data were extracted from the Survey of Medical Institutions. The number of care managers per 1000 people requiring long-term care and the number of care manager offices per 100 km^2^ of habitable area were used to determine the level of resources in a community. The former was calculated using the number of care managers in the prefecture in 2020 from the Survey of Institutions and Establishments for Long-term Care (Government Statistics Code: 00450042). The number of people requiring long-term care in the prefecture in 2020 was retrieved from the Report Survey on the Situation of Long-term Care Insurance Service (Government Statistics Code: 00450351). The latter was calculated using the number of care manager offices in each prefecture in 2020 from the Survey of Institutions and Establishments for long-term care. The habitable area of the prefecture in 2020 was determined by referencing the System of Social and Demographic Statistics (Government Statistics Code: 00200502). The proportion of hospitals providing patients with electronic medical information was used to determine the use of electronic health records, and it was calculated using the number of hospitals providing electronic medical information to patients in the prefecture in 2020. Moreover, the number of hospitals in the prefecture in 2020 was derived from the Survey of Medical Institutions. In the survey, for a case to be counted as providing patients with electronic medical information, this information had to be made available on Compact Disc-Recordable or an internet-based platform so that it could be imported into other medical systems for future use. Furthermore, the proportion of older adults living alone and average income per capita in the prefecture were also used.

### Statistical Analysis

SCR and supply-adjusted SCR were summarized using descriptive statistics to evaluate and compare the frequency of the provision of posthospitalization care planning across 47 prefectures. The mean and median values, SD, IQR, and the ratio of minimum to maximum were calculated.

Multivariate negative binomial regression models were used. Initially, we assumed a Poisson distribution because the distribution of each outcome was skewed. However, we suspected overdispersion during the dispersion test. A negative binomial distribution does not assume that the variance equals the mean, allowing for more overdispersion [25,26]. Four separate negative binomial regression models were developed. The dependent variables were (1) the number of patients who were discharged from the hospital to home, (2) the number of patients readmitted within 30 days after discharge from the hospital or clinic, (3) average length of hospital stay, and (4) medical expenditure in each prefecture in 2020. The independent variables included supply-adjusted SCR, with socioeconomic factors as covariates, specifically the proportion of older adults living alone and average per capita income. Supply-adjusted SCR was standardized to a *z*-scale to simplify interpretation [26]. The total number of patients discharged from the hospital, the total number of patients discharged from the hospital or clinic, and the population of each prefecture in 2020 were included as offset terms in each model. They were extracted from the Patient Survey and the Estimates of National Medical Care Expenditures. The absolute changes in each outcome were calculated according to the results of the multivariate negative binomial regression models.

The following steps were undertaken to investigate prefectural factors that may account for regional differences in the provision of posthospitalization care planning. The 47 prefectures were divided into 3 groups (low, medium, and high) based on the tertiles of each potential factor explaining regional differences in the provision of posthospitalization care planning. Subsequently, the supply-adjusted SCRs were compared among the 3 groups for each factor using the Mann-Whitney *U* test, with no adjustment for pairwise comparisons [27].

Statistical significance was set at a 2-sided *P* value of less than .05. All statistical analyses were performed using R (R Core Team) for Mac.

## Results

### Regional Differences in the Provision of Posthospitalization Care Planning

The summarized SCRs and supply-adjusted SCRs are shown in Table 1. The median value (IQR) of the SCRs was 102.81 (81.01-123.9). The minimum SCR was 17.07, which was recorded in Aomori Prefecture in the northern part of Japan, and the maximum was 181.45, which was recorded in Okinawa Prefecture in the southern part of Japan. The maximum-to-minimum ratio was 10.63. The median value (IQR) of supply-adjusted SCRs was 0.094 (0.07-0.116). The minimum value was 0.015, which was found in Aomori Prefecture. The maximum was 0.174 in Okinawa Prefecture. The maximum-to-minimum ratio was 11.60.

SCRs and supply-adjusted SCRs were calculated based on the number of claims for NSK1, an incentive program for hospital discharge planning that includes posthospitalization care planning, for each prefecture in Japan in 2020. Higher SCR and supply-adjusted SCR values indicate a more frequent provision of posthospitalization care planning services.

**Table 1:** Summary of SCRs^a^ and supply-adjusted SCRs for posthospitalization care planning.

	Total number of claims for NSK1^b^ (n=3,139,709; from 47 prefectures)	
	SCR (100 represents equal to all of Japan)	Supply-adjusted SCR
Value, median (IQR)	102.82 (81.01-123.9)	0.094 (0.07-0.116)
Value, mean (SD)	100.33 (31.92)	0.093 (0.034)
Value, minimum (prefecture)	17.07 (Aomori)	0.015 (Aomori)
Value, maximum (prefecture)	181.45 (Okinawa)	0.174 (Okinawa)
Value, ratio of minimum to maximum	10.63	11.60

^a^SCR: standardized claim ratios.

^b^NSK1: Nyutaiin Shien Kasan No. 1.

### Standardized Claim Ratio

**Figure 1:**
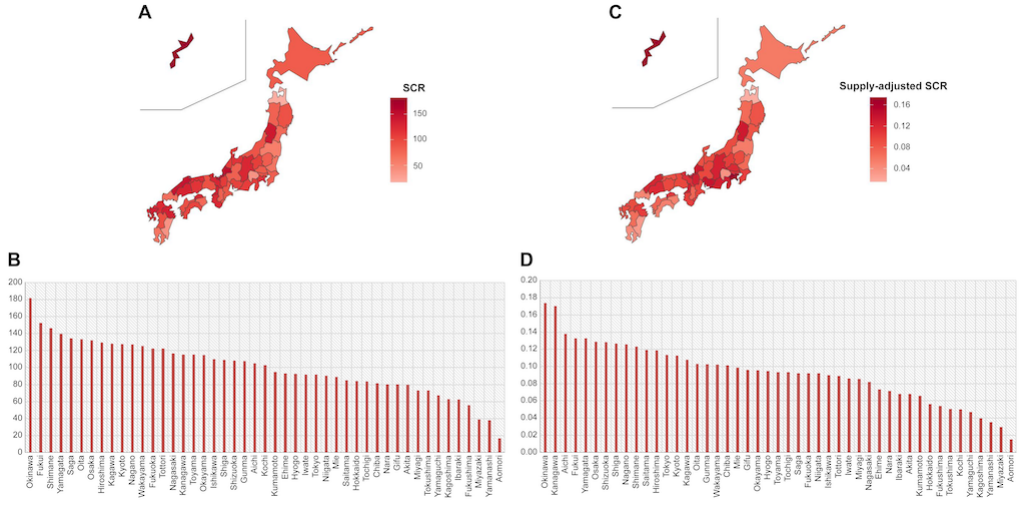
Regional differences in supply-adjusted SCRs for posthospitalization care planning claims in Japan during 2020, illustrating disparities related to health care resource distribution. (A) Maps representing regional differences by plotting SCRs on 47 prefectures. (B) Bar graph displaying SCRs of 47 prefectures arranged in decreasing order. (C) Maps representing regional differences by plotting supply-adjusted SCRs of 47 prefectures. (D) Bar graph of supply-adjusted SCRs of 47 prefectures arranged in decreasing order. SCRs and supply-adjusted SCRs were calculated based on the number of claims for Nyutaiin Shien Kasan No. 1, an incentive program for hospital discharge planning that includes posthospitalization care planning, for each prefecture in Japan in 2020. Higher SCR and supply-adjusted SCR values indicate a more frequent provision of posthospitalization care planning services. SCR: standardized claim ratio.

**Figure 2:**
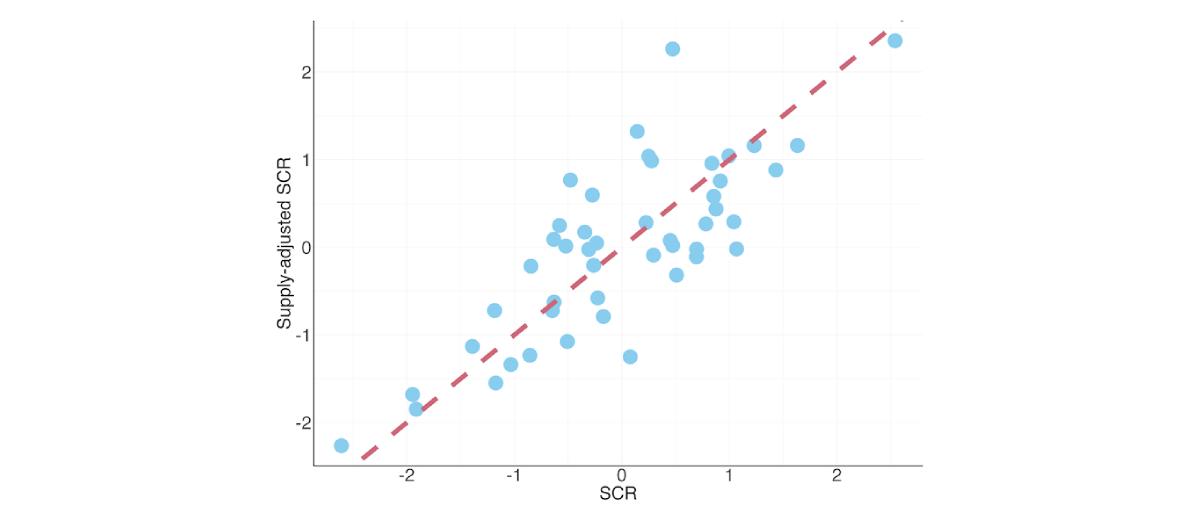
Scatter plots show the relationship between SCRs and supply-adjusted SCRs. SCRs and supply-adjusted SCRs were calculated based on the number of claims for Nyutaiin Shien Kasan No. 1, an incentive program for hospital discharge planning that includes posthospitalization care planning, for each Japanese prefecture in 2020. Both values were standardized to a z-scale to facilitate direct comparison. SCR: standardized claim ratio.

The maps and graphs visually illustrate regional differences (Figure 1A-D). As shown in Figure 2, the *z* score of the supply-adjusted SCR did not perfectly align with the *z* score of the SCR within a prefecture, which may suggest that SCRs are related to health care supply in each prefecture. Therefore, we used supply-adjusted SCRs for the subsequent statistical analyses.

### Association of Outcomes With Supply-Adjusted SCRs for Posthospitalization Care Planning

**Figure 3:**
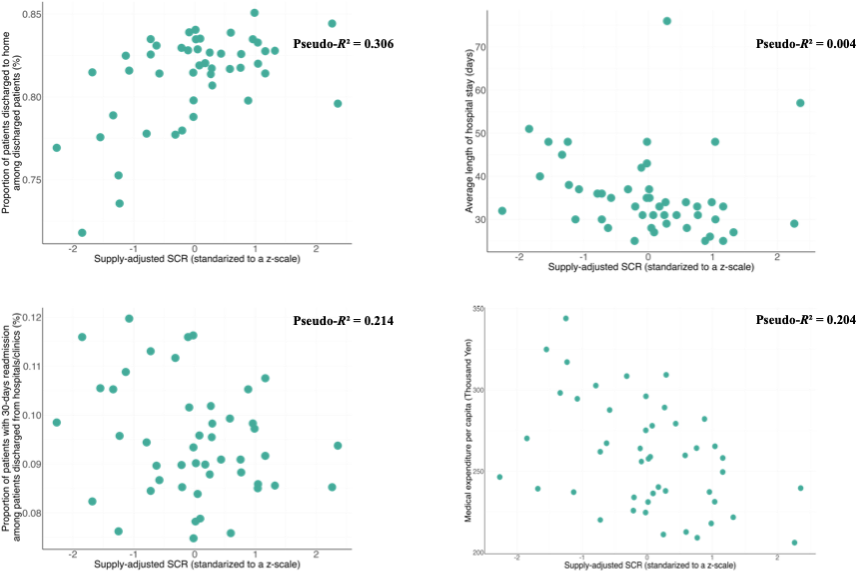
Scatter plots depicting bivariate associations between health care outcomes and supply-adjusted SCRs in an ecological study of 47 Japanese prefectures, based on 2020 data. The use of a z-scale for supply-adjusted SCRs allows for easier interpretation of the associations. SCR: standardized claim ratio.

The bivariate associations of the outcomes with supply-adjusted SCRs are presented as scatter plots and as unadjusted ratios resulting from the negative binomial regression models (refer to Figure 3 and Table 2). The supply-adjusted SCR was significantly associated with the outcomes (all *P*<.05), except for the 30-day readmission after discharge from hospital or clinic (*P*=.07) and average length of hospital stay (*P*=.20), in the unadjusted models.

**Table 2:** Association of outcomes with supply-adjusted standardized claim ratios for posthospitalization care planning^a^. Results from bivariate and multivariate negative binomial regression analyses in an ecological study of 47 prefectures in Japan, based on 2020 data.

Outcome	Unadjusted		Adjusted	
	Ratio (95% CI)	*P* value	Ratio^b^ (95% CI)	*P* value
Discharge from hospital to home	1.02 (1.01-1.03)	<.001	1.01 (1.00-1.02)	.03
30-day readmission after discharge from the hospital or clinic	0.97 (0.94-1.00)	.07	0.99 (0.95-1.03)	.50
Average length of hospital stays	0.96 (0.89-1.03)	.20	1.02 (0.94-1.10)	.70
Medical expenditure per capita	0.94 (0.91-0.97)	<.001	1.00 (0.98-1.03)	>.99

^a^Supply-adjusted standardized claim ratios were standardized to a *z*-scale.

^b^Adjusted for the prevalence of older people living alone and the average income per capita in each prefecture.

Multivariate associations of outcomes with supply-adjusted SCRs are presented as adjusted ratios in Table 2. The supply-adjusted SCRs were significantly associated with discharge from hospital to home (*P*=.03). This may indicate that when supply-adjusted SCR increases by 1 SD, the number of patients discharged to home increases by 9.68 (95% CI 0.98-18.47) per 1000 patients discharged from the hospital, as shown in Table 3. Absolute change in each outcome is associated with a 1-SD increase in supply-adjusted SCRs. It was calculated based on the results of multivariate negative binomial regression analyses in an ecological study of 47 prefectures in Japan, based on 2020 data.

**Table 3:** Absolute change in each outcome when supply-adjusted standardized claim ratios increase by 1 SD. Absolute change in each outcome is associated with a 1-SD increase in supply-adjusted SCRs. It was calculated based on the results of multivariate negative binomial regression analyses.

Outcomes	Absolute change^a^ (95% CI)
Discharge from hospital to home (change in number of patients per 1000 patients who are discharged from hospital)	9.68 (0.98 to 18.47)
30-day readmission after discharge from hospital or clinic (change in the number of patients per 1000 patients who are discharged from hospital or clinic)	–12.47 (–48.58 to 25)
Average length of hospital stays (change in days of length of hospital stay)	0.67 (–1.92 to 3.11)
Medical expenditure per capita (change in US $ [JP ¥^b^] of medical expenditure per capita)	2.24 [335.78] (−40.09 [−6000.36] to 45.64 [6831.14])

^a^Adjusted for the prevalence of older people living alone and average income per capita in each prefecture.

^b^US $ 1=JP ¥149.67.

### Factors Explaining Regional Differences in the Provision of Posthospitalization Care Planning

**Figure 4:**
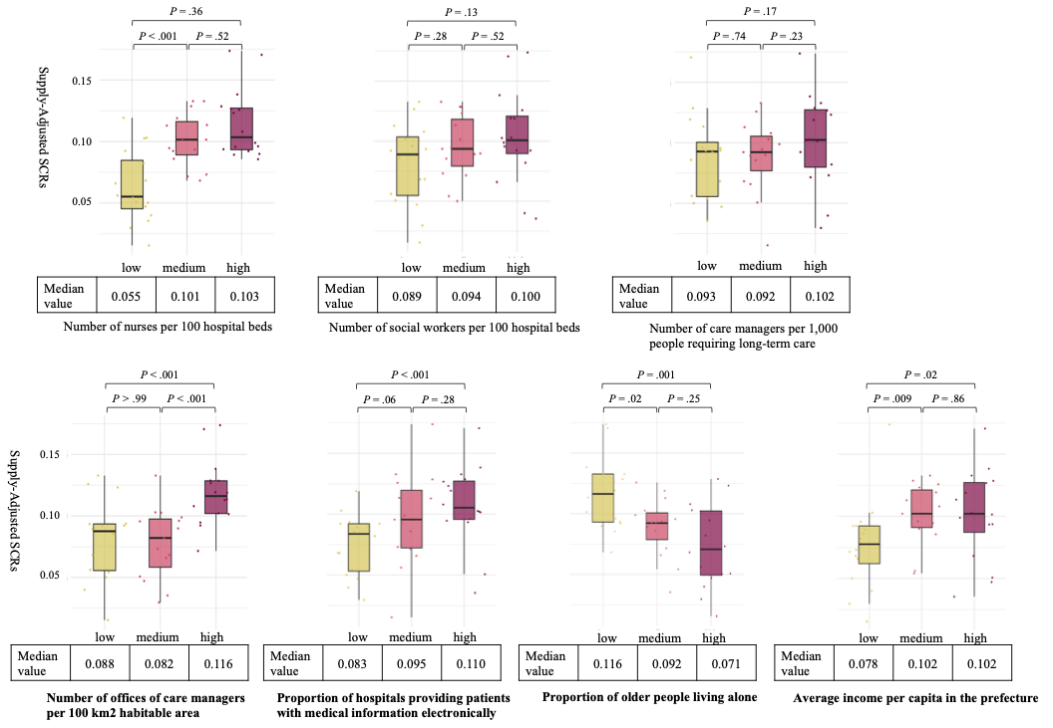
Box plots representing comparisons of supply-adjusted SCRs among the 3 groups based on each factor. The 47 Japanese prefectures were divided into tertiles based on each contributing factor. Box plots illustrate differences in supply-adjusted SCRs among these groups, with comparisons analyzed using the Mann-Whitney U test, based on 2020 data from this ecological study. SCR: standardized claim ratio.

The results of comparing the 3 groups for each factor using the Mann-Whitney *U* test are shown in Figure 4. A greater supply-adjusted SCR for posthospitalization care planning was significantly associated with a higher number of nurses per 100 hospital beds, a higher number of offices of care managers per 100 km^2^ of habitable area, a higher proportion of hospitals providing patients with electronic medical information, a lower proportion of older adults living alone, and a higher average income per capita in the prefecture.

## Discussion

### Principal Findings

This ecological study found that posthospitalization care planning was associated with discharge to home at the prefectural level. In addition, we identified regional disparities in the provision of posthospitalization care planning across Japanese 47 prefectures. These disparities were explained by the number of hospital nurses, the number of care manager offices, and the utilization rate of electronic medical information in hospitals within each prefecture.

### Limitations

This study has several limitations. First, as an ecological study, it is susceptible to ecological fallacy, and confounder adjustment may not have been sufficient due to unmeasured confounders, particularly at the individual level. Therefore, our results cannot be directly applied to individual-level scenarios. Second, both SCR and supply-adjusted SCR reflect the number of claims rather than the actual extent of posthospitalization care planning efforts across all hospitals in Japan. Some hospitals may provide such efforts voluntarily without submitting a claim. In prefectures with a higher proportion of these hospitals, SCR and supply-adjusted SCR may underestimate the true level of posthospitalization care planning efforts. Third, our exposure and outcome measures might have been influenced by disease patterns and severity within each prefecture, which we did not account for in this study.

In addition, the data used for this study were primarily collected during the COVID-19 pandemic. A study conducted on acute care hospitals in Japan reported that discharge destination trends during the pandemic did not change significantly compared with prepandemic trends [28]. However, patient health care–seeking behavior changed markedly between the pre- and post-pandemic periods [29,30], potentially impacting health care service delivery and resource allocation. While the full extent of the pandemic’s impact remains unclear, we recognized that these shifts in health care delivery and resource allocation present limitations when applying prepandemic data to the current context. Therefore, we deemed it reasonable to use the most recent postpandemic data. The Patient Survey 2020, which we used for outcome variables, represents the latest available data following the pandemic. However, to fully examine the association between exposure and outcomes, future studies using data beyond 2020 will be essential for further validation.

Despite these limitations, this is the first nationwide study to explore the association between posthospitalization care planning and outcome measures in Japan using NDB open data. Our findings provide valuable insights that can inform the selection of outcome measures in future studies evaluating the impact of health care policies. In addition, this study highlights regional disparities in the accessibility of posthospitalization care planning services under health insurance and related factors, offering fundamental knowledge on regional inequities in hospital care in Japan, a super-aged society facing growing social inequalities.

### Comparison With Previous Work

Our results show that a higher number of claims for NSK1, indicating more frequent provision of posthospitalization care planning, is associated with a greater number of patients being discharged to home. This finding suggests that posthospitalization care planning may facilitate patient discharge from the hospital to home, particularly for those with complex needs, who are specifically targeted by NSK1. Skovgaard et al [31] documented that discharging patients with complex needs is a multifaceted process that requires careful coordination and communication among the patient, their family members, and health care providers in the community to ensure discharge readiness and the availability of care infrastructure after discharge. Other studies have similarly emphasized that involving patients and their relatives in coordinating postdischarge care services is essential for effective discharge planning and transitional care [6,32-34]. The standardized process of the incentive for NSK1 mandates the creation of a discharge plan, the organization of meetings, and the inclusion of the patients, their families, and multidisciplinary providers early in the hospitalization phase. It also requires communication with long-term care and social care providers to ensure seamless posthospitalization care planning. Based on this, it can be hypothesized that NSK1, as a financial incentive, could contribute to ensuring discharge readiness for patients and their families and establishing the necessary care infrastructure, thereby promoting discharge to home. Although previous studies have rarely examined this in detail, discharge to home is critical for “aging in place” and should be regarded as an important outcome measure of NSK1 in future research using detailed data.

Our results highlight regional variation in the number of claims for NSK1 across Japan, which may reflect disparities in the accessibility of posthospitalization care planning services under health insurance across prefectures. More frequent provision of these services is associated with higher average per capita income in the prefecture. Given Japan’s universal health coverage and medical assistance system, it is unlikely that patients living in poverty are excluded from receiving posthospitalization care planning services due to economic hardship. However, since the quality of hospital care may be influenced by geographic location [35], our findings suggest that care quality may be limited in areas with lower socioeconomic status.

In addition, our results indicate that a more frequent provision of posthospitalization care planning is associated with a higher number of nurses per 100 hospital beds. The MHLW has reported that a shortage of human resources, such as nurses and social workers in hospitals, is a major barrier to the fulfillment of NSK1 requirements [22]. Indeed, Japan faces a regional maldistribution of health care resources, including physicians, nurses, and rehabilitation therapists [36-38]. This study also found that the density of care manager offices per 100 km² of habitable area is associated with regional variation in the provision of posthospitalization care planning. This finding underscores the importance of not only hospital-based health care resources but also community-based long-term care resources in the provision of posthospitalization care planning. For example, hospital staff can more easily establish relationships and communicate with care managers when their offices are located near the hospitals. The availability of digitized medical information is another crucial factor. Digitalization is essential for electronic information sharing, which is recognized as a key facilitator of care coordination and continuity of care [23] and is likely associated with optimal patient outcomes [24]. However, in Japan, the adoption of the electronic medical record (EMR) system, which serves as the foundation for digital medical information, has been limited [39,40]. The Japanese government currently provides financial assistance to hospitals seeking to adopt the EMR system, with the long-term goal of establishing a national medical information platform to consolidate health care–related data. These policy efforts, by helping to mitigate the impact of resource shortages and enhancing efficiency in care coordination, including communication between health care and long-term care providers, may help reduce regional disparities in the number of claims for NSK1. Our findings support the need for such ongoing initiatives to enhance the effectiveness of posthospitalization care planning nationwide.

### Conclusions

Our data analysis of nationwide health claims suggested that early care coordination might facilitate discharge from hospital to home. However, regional disparities in accessibility were found. Our findings underline the importance of addressing regional disparities in health care resources to promote equitable outcomes. Further research drawing on detailed data is needed to clarify the mechanism of causality.

## Abbreviations

EMR: electronic medical record
MHLW: Ministry of Health, Labour and Welfare
NDB: National Database of Health Insurance Claims and Specific Health Checkups
NSK1: Nyutaiin Shien Kasan No. 1
SCR: standardized claim ratio
STROBE: Strengthening the Reporting of Observational Studies in Epidemiology

## Data Availability

All data analyzed during this study are publicly accessible as open-source data [42-50].
